# Warfarin Patient Self-Management in the US Health Care System: A Nonrandomized Clinical Trial

**DOI:** 10.1001/jamanetworkopen.2026.2627

**Published:** 2026-03-19

**Authors:** Daniel M. Witt, Heeseung Hong, Aaron S. Wilson, Aubrey E. Jones, Sara R. Vazquez, Spencer J. Gilbert, Daniel C. Malone, Nathorn Chaiyakunapruk, Jordan B. King, Geoffrey D. Barnes, Katelyn W. Sylvester, Linh Chan, Thomas Delate

**Affiliations:** 1 College of Pharmacy, University of Utah, Salt Lake City; 2 Health Thrombosis Service, University of Utah, Salt Lake City; 3 IDEAS Center, Veterans Affairs Salt Lake City Healthcare System, Salt Lake City, Utah; 4 School of Medicine, University of Utah, Salt Lake City; 5 Institute for Health Research, Kaiser Permanente Colorado, Aurora, Colorado; 6 University of Michigan, Ann Arbor; 7 Brigham and Women’s Hospital, Boston, Massachusetts; 8 Veterans Affairs Loma Linda Healthcare System, Loma Linda, California; 9 Kaiser Permanente National Pharmacy, Aurora, Colorado

## Abstract

**Importance:**

Warfarin patient self-management (PSM) has consistently demonstrated superior efficacy to clinic-based management and is recommended by evidence-based guidelines, but it is virtually unused in the US health care system.

**Objective:**

To implement and evaluate warfarin PSM in the US health care system using strategies developed to overcome previously identified barriers associated with PSM underutilization.

**Design, Setting, and Participants:**

This was a single-group implementation open-label trial conducted between March 2023 and January 2024 at 4 specialized anticoagulation management services facilities in the US. Patients who participated had been taking warfarin for at least 9 months and were using home international normalized ratio monitors. The follow-up period was 6 months. Data were analyzed between July and December 2025.

**Intervention:**

Transition to warfarin PSM using an implementation toolkit developed to address barriers specific to the US health care system.

**Main Outcomes and Measures:**

Implementation and clinical outcomes were assessed using the Reach, Effectiveness, Adoption, Implementation, Maintenance (RE-AIM) framework and included the proportion of participants successfully transitioned to PSM, time in the therapeutic international normalized ratio range, bleeding and thromboembolic events, and proportion of international normalized ratios managed independently by participants.

**Results:**

Of the 255 patients invited to participate in the trial, 138 (54.0%) consented (mean [SD] age, 63.2 [13.2] years; 80 male [58.0%]; 117 White [84.8%]; 123 non-Hispanic or Latino [89.1%]); 127 participants (92.0%) had been receiving warfarin therapy for 2 or more years. Among participants, 120 (87.0%) successfully transitioned to PSM and completed 6 months of follow-up. Compared with baseline, time in therapeutic range during PSM improved from 77.1% (95% CI, 73.7%-80.6%) to 81.3% (95% CI, 78.1%-84.4%). During the PSM phase, 5 bleeding events and no thromboembolic events or deaths occurred. Differences in adverse events in the 6 months before and after PSM initiation were not statistically significant. Most (83.0%) warfarin dosing decisions were made independently by participants and rarely required intervention from clinicians due to safety concerns. At the conclusion of the study, 98 participants (84.4%) expressed a preference for continuing PSM and clinicians expressed comfort with 109 participants (94.0%) continuing PSM.

**Conclusions and Relevance:**

In this single-group implementation trial, warfarin PSM was safely and effectively implemented. These results provide critically needed evidence to facilitate and accelerate PSM implementation in the US health care system.

**Trial Registration:**

ClinicalTrials.gov Identifier: NCT04766216

## Key Points

**Question:**

Can patients in the US health care system successfully transition to warfarin patient self-management (PSM)?

**Findings:**

In this single-group implementation trial including 138 participants, 87% were able to successfully transition to PSM. Time in the therapeutic international normalized ratio range improved during PSM and adverse events were similar to baseline.

**Meaning:**

These results suggest that warfarin PSM can be safely and effectively implemented within the US health care system.

## Teaser

This nonrandomized clinical trial examines outcomes of patients transitioning to warfarin self-management using an implementation toolkit developed to address barriers specific to the US health care system.

## Introduction

Despite the development of direct oral anticoagulants, warfarin remains the first-line oral anticoagulant for millions of people with mechanical heart valves,^1^ moderate-to-severe mitral stenosis associated with atrial fibrillation,^2^ antiphospholipid antibody syndrome,^3^ and left ventricular assist devices.^4^ Thus, warfarin therapy will remain a vital oral anticoagulant therapy option for the foreseeable future for many patients.

Warfarin is a narrow therapeutic index drug with variable interpatient dose response, pharmacogenetic variations, and numerous drug and dietary interactions.^5^ This variability necessitates attention to patient education and engagement, frequent monitoring of the intensity of anticoagulation using the international normalized ratio (INR), and subsequent warfarin dose adjustments to maintain the INR within a preestablished therapeutic range. Currently, most warfarin management is accomplished using INRs measured in laboratories or clinics, with warfarin doses determined by clinicians. This current process often leads to suboptimal warfarin management, increasing the risk of thromboembolic or bleeding events and is further complicated by social determinants of health such as limited transportation access and inadequate social support.^6,7^

Warfarin patient self-management (PSM) occurs when patients obtain their INR result and adjust their dose without receiving instructions from a clinician. Patients performing PSM often test their INR at home using portable INR testing devices, which further increases patient autonomy. Patients with diabetes have been using a similar process to self-manage blood glucose for decades.^8^ Clinical trials of PSM, conducted predominantly in Europe, have demonstrated the efficacy of PSM compared with clinician-based care strategies.^9^ In a systematic review of approximately 3500 PSM patients from 11 clinical trials, PSM was associated with improved INR control and reduced thromboembolic complications compared with clinic-based management.^10^ PSM was also associated with improved treatment satisfaction and decreased daily hassles, psychological distress, and strain on social networks.^11^ Based on these studies, guidelines from the American College of Chest Physicians, American Society of Hematology, and European Society of Cardiology recommend PSM over all other warfarin management strategies.^11,12,13^

In the US, patients receiving warfarin therapy are rarely offered PSM despite its being less burdensome, less expensive, and potentially safer than clinic-based warfarin management.^9,10^ We previously explored PSM barriers and facilitators from the perspectives of US clinicians and patients.^14,15^ Clinician concern for patient safety and uncertainty about patients’ ability to participate in PSM contribute to anticoagulation specialist hesitancy to relinquish control of warfarin monitoring.^15^ In contrast, we found that patients were willing and interested in trying PSM if a framework to support independent decision-making was present, and strong clinician-patient relationships were maintained as a safety net.^14^ Both clinicians and patients believed access to home INR monitors, previous experience with warfarin therapy, and proper training were important PSM facilitators.^13,14^ The objectives of this study were to develop and evaluate warfarin PSM implementation strategies in US ambulatory care sites—using previously identified barriers and leveraging identified facilitators—and to assess both implementation and clinical outcomes.

## Methods

### Study Design and Setting

Guiding frameworks and methods for this open-label study have been described in detail previously.^16^ Briefly, PSM implementation strategies were guided by the Consolidated Framework for Implementation Research (CFIR),^17^ the Quality Implementation Framework (QIF),^18^ and Rapid Cycle Research Methodology (RCRM).^19^ Using these frameworks, an organized structure to oversee the PSM implementation process was developed by the study team. A type III implementation-effectiveness hybrid research design was used, which allowed evaluation of implementation strategy outcomes while observing and gathering information on PSM’s impact on relevant clinical outcomes (trial protocol available in Supplement 2).^20^ The study was reviewed and approved by the institutional review boards of all participating sites, and each site obtained written informed consent from participants. Reporting of this study follows the Transparent Reporting of Evaluations With Nonrandomized Designs (TREND) Reporting Checklist.

Four anticoagulation management services recognized by the Anticoagulation Forum as Anticoagulation Centers of Excellence (ACE) served as enrollment sites for this single-arm implementation trial. The ACE program provides a structured approach to achieving consistent and sustainable excellence in anticoagulation therapy management. Study sites included the University of Michigan (staffed by nurses), the University of Utah, Brigham and Women’s Hospital, and the Veterans Administration Loma Linda Health System (all staffed by pharmacists).

### PSM Implementation Toolkit Development

Workgroups consisting of 3 to 5 study team members met regularly over a period of several months to develop a warfarin PSM implementation toolkit (see eMethods in Supplement 1). The implementation toolkit included information pertaining to patient selection criteria, PSM educational tools and knowledge assessments, approaches for documenting PSM activities in the electronic health record (EHR), and clinical decision support tools.

#### Patient Selection Criteria

General PSM patient selection criteria included medical stability at the time of PSM initiation (clinicians considered overall stability and readiness for PSM; recent hospitalizations or emergency department visits could preclude study participation if they indicated clinical instability or increased risk); cognitive stability and ability to follow instructions and/or support from a willing and capable caregiver; and current home INR monitor use. Specific PSM patient selection criteria related to participation in the PSM implementation study included goal INR range 2.0 to 3.0 or 2.5 to 3.5; at least 9 months of prior warfarin therapy at time of enrollment; use of a single warfarin tablet strength; at least an additional 6 months warfarin therapy planned; willingness to test INR every 7 to 14 days; receiving majority of care within the enrolling site’s health care system; and ability to maintain internet access throughout study participation.

#### PSM Educational Tools and Knowledge Assessment

Participants received PSM educational tools and knowledge assessments via an internally developed interactive online module. Participants completed a prestudy PSM competency assessment and were asked to indicate their PSM comfort level (very uncomfortable, somewhat uncomfortable, somewhat comfortable, very comfortable) upon completion of the module. Raw competency scores were shared with clinicians to inform decisions about continued study participation; however, no predefined passing score was used for this determination.

#### Approaches for Documenting PSM Activities in the EHR

During PSM, participants self-reported the following using an online survey hosted in the Research Electronic Data Capture (REDCap) software platform^21,22^: INR results, factors potentially influencing INR results, warfarin dosing decision details, and any bleeding or thromboembolic events. After each INR, a summary of the REDCap survey was emailed directly to site clinicians for review, enabling timely intervention in potentially harmful situations and assessment of continued study participation. PSM INR results were documented in the EHR the same day.

#### Clinical Decision Support Tools

Participants received PSM clinical decision tools, including an online warfarin dosing tool and manual algorithms. Participants or their caregivers were also allowed to make warfarin dosing decisions based on their own experience without using formal clinical decision support tools if desired. Participants were instructed to contact anticoagulation clinicians in high-risk situations (eg, INR below 1.5 or above 5.0, symptoms of bleeding or thromboembolism, or prior to scheduled invasive procedures) or if they wished to consult with a clinician for any reason.

### PSM Implementation

The 5 elements of the Reach, Effectiveness, Adoption, Implementation, Maintenance (RE-AIM) framework were used for assessing implementation and effectiveness outcomes.^23^ Each enrolling site recruited English-speaking patients at least 18 years of age who met the previously defined patient selection criteria. Each site consented and enrolled eligible patients, tracked PSM performance, and prospectively collected outcome data using online REDCap forms hosted at the University of Utah.

#### Recruitment Process

During usual patient care activities, clinicians asked patients who met inclusion criteria if they would be interested in participating in the PSM implementation study. Those expressing interest were asked the following screening questions to determine suitability for study participation: Why are you taking warfarin? What is your INR goal range? What is your warfarin tablet strength? What would you do with your warfarin dose if your INR result was too low? What bad things could potentially happen if your INR gets too high? Patients providing satisfactory responses to the screening questions were referred to site study coordinators to discuss further participation in the study and complete the written consent process. Enrollment occurred between March 1, 2023, and January 31, 2024. Baseline characteristics including age, self-reported sex category, self-reported race and ethnicity categories, and primary indication for warfarin therapy were abstracted from the EHR. Participants received a modest monetary incentive after completion of baseline and end of study surveys.

#### PSM Implementation Phase

Participants received PSM training using implementation toolkit materials, including the previously described interactive online module, to prepare for PSM initiation. At each site, clinician study team members evaluated each participant using 3 criteria: raw competency assessment score, stated comfort level with PSM, and the clinicians’ subjective assessment of the participant’s likelihood of success. Based on this evaluation, clinicians decided whether the participant should progress to the *spotters ready* phase, a structured wash-in period during which clinicians provided feedback on the participant’s dosing plans as needed. The spotters ready phase ended and the formal PSM phase began once participants expressed feeling comfortable starting PSM on their own.

During the 6-month PSM phase, participants were asked to check INRs every 1 to 2 weeks when INRs were in range and weekly when INRs were out of range. Participants were asked to use clinical decision support tools from the implementation toolkit or their own judgment to make independent warfarin dosing decisions. Participants were asked to contact clinicians in high-risk situations as previously described. Using the REDCap online form, participants self-reported INR results, warfarin dosing decisions and methods, factors potentially affecting INRs (eg, medication, diet, health changes), and any bleeding or thromboembolic events.

### Outcomes

#### Reach

Prior to initiating PSM, participants completed an online REDCap survey containing the following: (1) the 28-item Anticoagulation Knowledge Tool (AKT)^24^; (2) the 15-item Anti-Clot Treatment Scale (ACTS) for assessing satisfaction with anticoagulant treatment that includes 12-item Burdens and 3-item Benefits scales^25^; and (3) the Rand Short Form 36 questionnaire (SF-36) for assessing health-related quality of life.^26^ The survey also asked participants to specify current duration of warfarin therapy (less than 2 years, greater or equal to 2 years). The percentage of participants successfully transitioned to PSM was calculated using the following denominators: (1) all patients receiving warfarin; (2) all patients invited to participate in the PSM study, including those who declined participation; and (3) all participants who provided informed consent and attempted transitioning to PSM (primary implementation outcome). Successful transition to PSM was defined as completing 6 months of PSM. Reasons participants were unable to successfully transition to PSM during implementation efforts were recorded. At the end of the PSM phase, participants were asked to complete a final survey consisting of the same questions as the initial survey (AKT, ACT, and SF-36).

#### Effectiveness

For participants who successfully transitioned to PSM, the INR control outcomes that were compared between the 6-month periods before and after PSM were (1) individual time in the therapeutic INR range (TTR) using linear interpolation^27^ and (2) proportion of INRs resulting in a warfarin dose change. Additional effectiveness outcome comparisons before and after PSM transition included major bleeding and clinically relevant nonmajor bleeding events as defined by the International Society on Thrombosis and Hemostasis,^28,29^ thromboembolic events, and all-cause mortality. Thromboembolic events included stroke, systemic embolism, or venous thromboembolism documented by objective imaging. Bleeding and thromboembolic events were confirmed by manual EHR review.

#### Adoption

Characteristics associated with willingness to participate in the PSM implementation study were entered into multivariable logistic regression models to identify variables that were independently associated with willingness to participate.

#### Implementation (Fidelity)

The percentage of INRs independently managed by participants (ie, the participant used their INR to determine a warfarin dosing plan without contacting a clinician) were calculated. The proportions of in- and out-of-range INRs that did not result in a warfarin dose change were also calculated. During the post-PSM phase survey, participants were asked to rate the provided warfarin dosing decision tools as very useful, useful, or not useful.

#### Maintenance

At the end of follow up, participants who successfully transitioned to PSM were asked to complete a brief online survey where they responded to the following question, “Would you prefer to continue managing your own warfarin therapy?” (yes, no, or unsure) and were given the opportunity to provide reasons for their answer. Clinicians at each site were asked a similar question, “Are you comfortable with this participant continuing PSM?” (yes, no, unsure) and given the opportunity to provide reasons for their answer.

### Statistical Analysis

Using a 1-sample *t* test for sample size estimation, we determined that 125 participants would be required to detect an absolute change of 4.5% in TTR during PSM, with 80% power and a 2-sided α = .05, assuming a standard deviation of 18.0%. To accommodate potential loss to follow-up and failure to transition to PSM, we targeted an enrollment of 150 participants. Given the established link between higher TTR and improved clinical outcomes,^30,31,32^ a 4.5% increase in an already well-controlled cohort was deemed clinically meaningful.

For participants who successfully transitioned to PSM, AKT, ACT, and SF-36 scores were summarized descriptively and compared pre- and posttransition. Data for reach, effectiveness, and implementation outcomes were summarized descriptively with the Shapiro-Wilk test for normality utilized to assess the distributions of continuous data. Due to nonnormality of the AKT, ACT, and SF-36 scores, data were analyzed with both parametric and nonparametric tests. Outliers were assessed based on 99% upper and lower limits. Multilevel linear and logistic models were used for continuous and binary outcomes, respectively, with nesting by site. These models compared differences in continuous and categorical outcomes between patients who did and did not participate in the PSM implementation phase, who did and did not successfully transition to PSM, and among patients who successfully transitioned, evaluated changes in outcomes before and after transition. *P* < .05 was considered statistically significant in 2-sided tests.

The comparator group for the multivariable logistic regression model for the “adoption” outcome was patients who met study inclusion criteria but declined study participation. Collected variables included age, gender, race, ethnicity, and indication(s) for warfarin therapy.

Maintenance outcomes, including patient preferences and clinician comfort with continuing PSM poststudy, were summarized as proportions. Statistical analyses were performed using PC-SAS version 9.4 (SAS Institute Inc).

## Results

**Figure 1.**
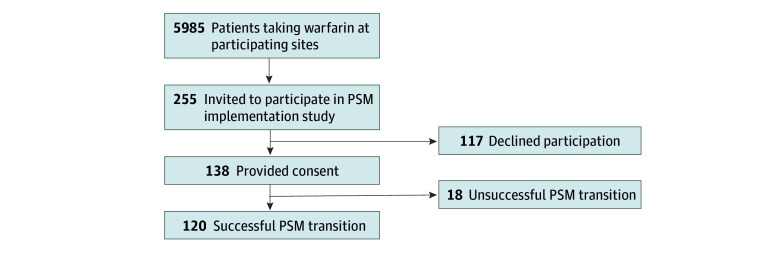
Study Flow Diagram PSM indicates patient self-management.

There were 5985 patients receiving warfarin across the 4 study sites at the beginning of PSM recruitment (1648 at the University of Utah, 1775 at Brigham and Women’s Hospital, 2357 at the University of Michigan, and 205 at the Veterans Administration Loma Linda Healthcare system) (Figure 1). A total of 255 patients (4.3%) were invited to participate in the PSM implementation phase of the study; 138 of those (54.0%) consented to participate in the study (mean [SD] age, 63.2 [13.2] years; 80 male [58.0%]; 5 Black [3.6%], 117 White [84.8%]; 15 Hispanic or Latino ethnicity [10.9%]) (Table 1).

**Table 1.** Baseline Characteristics of Recruited Patients by Consent Status

Characteristic	Participants, No. (%) (N = 255)		*P* value
	Did not consent (n = 117)	Consented (n = 138)	
Mean age (SD), y^a^	68.5 (12.5)	63.2 (13.2)	.002
Sex			
Female	55 (47.0)	58 (42.0)	.43
Male	62 (53.0)	80 (58.0)	
Race			
American Indian or Alaska Native	1 (0.9)	1 (0.7)	.75
Asian	0	2 (1.5)	
Black or African American	5 (4.3)	5 (3.6)	
Multiracial	0	1 (0.7)	
Native Hawaiian or other Pacific Islander	0	1 (0.7)	
White	99 (84.6)	117 (84.8)	
Unknown or undeclared	7 (6.0)	5 (3.6)	
Other^b^	5 (4.3)	6 (4.4)	
Hispanic or Latino ethnicity	5 (4.3)	15 (10.9)	.05
Organization			
Brigham and Women’s	19 (16.2)	30 (21.7)	.01
Loma Linda	20 (17.1)	30 (21.7)	
Michigan	14 (12.0)	30 (21.7)	
Utah	64 (54.7)	48 (34.8)	
Primary indication for anticoagulation			
Antiphospholipid syndrome	4 (3.4)	9 (6.5)	.22
Atrial fibrillation/flutter	40 (34.2)	30 (21.7)	
Cerebral venous sinus thrombosis	0	2 (1.5)	
Cerebrovascular accident	2 (1.7)	1 (0.7)	
Mechanical heart valve	35 (29.9)	53 (38.4)	
Venous thromboembolism	33 (28.2)	39 (28.3)	
Other^c^	3 (2.6)	4 (2.9)	
Years taking warfarin			
<2	6 (5.1)	10 (7.3)	.78
≥2	110 (94.0)	127 (92.0)	
Unknown	1 (0.9)	1 (0.7)	

^a^ As of recruitment date.

^b^ Includes participants who selected other.

^c^ Includes hypercoagulable state, lupus anticoagulant, mural thrombus, peripheral artery disease, protein C deficiency, transient ischemic attack, Fontan procedure.

### Reach

Baseline characteristics of those who did and did not consent to participate in the PSM implementation phase were similar except that patients who did not consent were older than those who did (mean [SD] age: did not consent, 68.5 [12.5] years vs consented, 63.2 [13.2] years) (Table 1). Among consenting participants, 127 (92.0%) had been receiving warfarin therapy for 2 or more years, with the most common indications being 53 participants (38.4%) with mechanical heart valves, 39 (28.3%) with venous thromboembolism, and 30 (21.7%) with atrial fibrillation.

The mean (SD) baseline AKT score of 88.7 (10.6) did not change appreciably following 6 months of PSM (*P* = .51) (eTable 1 in Supplement 1). None of the changes from baseline ACTS or SF-36 scores at the end of the study were statistically significant. The mean (SD) educational module competency assessment score was 85.4% (12.0%), with 92.0% (115 of 125 who completed the online educational module) indicating they felt very comfortable trying PSM.

Of the 138 consenting participants, 120 (87.0%) successfully transitioned to PSM. This equated to 2.0% of the total warfarin-treated patients across all study sites. Of the 18 participants who did not successfully transition to PSM, 12 withdrew from the study before attempting PSM. Reasons for study withdrawal in the remaining 6 participants included moving out of the area, being overburdened by study procedures, and deciding PSM was not for them. Baseline characteristics of those who did and did not successfully transition to PSM were similar except for warfarin therapy indication (eTable 2 in Supplement 1).

### Effectiveness and Safety

**Figure 2.**
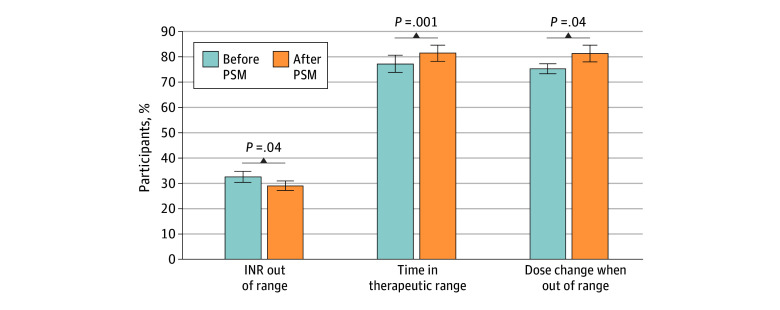
INR Control Outcomes in the 6 Months Before and After PSM Implementation Error bars denote the 95% CI. PSM indicates patient self-management; INR, international normalized ratio.

Compared with the 6 months preceding transition to PSM, TTR during the 6-month PSM phase improved significantly, from 77.1% (95% CI, 73.7%-80.6%) at baseline to 81.3% (95% CI, 78.1%-84.4%) for participants who successfully transitioned to PSM (*P* = .001) (Figure 2). The proportion of out-of-range INRs declined from 32.5% (95% CI, 30.4%-34.6%) to 29.0% (95% CI, 27.2%-31.0%) in the 6 months before and after PSM transition (*P* = .04). The proportion of out-of-range INR results that led to a warfarin dose change increased from 75.1% (461 of 614) to 81.2% (441 of 662) in the 6 months before and after PSM transition, respectively (*P* = .04). There were 3 major bleeding events, 2 clinically relevant nonmajor bleeding events, and no thromboembolic events or deaths during the PSM phase. Adverse event rates were not different between those who did and did not successfully transition to PSM (Table 2). Differences in adverse events in the 6 months before and after PSM initiation were also not statistically significant. The proportion of participants with emergency department visits or hospitalizations for any reason was numerically lower in those successfully transitioning to PSM, but this difference was not statistically significant (38.9% [7 of 18] vs 20.0% [24 of 120]; *P* = .10).

**Table 2.** Adverse Clinical Events in the 6 Months Before and After PSM Transition

Outcome	Events, No. (%)			*P* value^a^
	6 mo before transition (n = 138)	6 mo after transition		
		Unsuccessful PSM transition (n = 18)	Transitioned to PSM (n = 120)	
ED visit or hospitalization	31 (22.5)	7 (38.9)	24 (20.0)	.10
All-cause mortality	NA	0	0	>.99
Bleeding-related ED visit or hospitalization	3 (2.2)^b^	0	5 (4.2)^c^	.99
Major bleeding	0	0	3 (2.5)^d^	.99
Thrombosis-related ED visit or hospitalization	0	0	0	>.99

Abbreviations: PSM, patient self-management; ED, emergency department.

^a^ Comparison between 6 months after PSM transition groups; data were structured hierarchically to account for nesting by site.

^b^ Bleeding sites include skin, epistaxis, and genitourinary (1 event each).

^c^ Bleeding sites include gastrointestinal (3 events) and postsurgical procedure (2 events).

^d^ Among patients with a bleeding-related ER visit/hospitalization.

### Adoption

Logistic regression modeling of factors independently associated with willingness to participate in the PSM implementation study found that older patients were less likely to agree to participate (adjusted odds ratio, 0.97; 95% CI, 0.95-0.99) (Table 3). Associations related to gender, race, ethnicity, and warfarin therapy indication were not statistically significant.

**Table 3.** Logistic Regression Modeling of Factors Independently Associated With Willingness to Participate in PSM Implementation Study

Characteristic	Adjusted OR (95% CI) (N = 255)^a^
Age^b^	0.97 (0.95-0.99)
Sex	
Male	1 [Reference]
Female	0.78 (0.44-1.41)
Hispanic/Latino ethnicity	1.49 (0.49-4.95)
Primary indication for anticoagulation	
Mechanical heart valve	1 [Reference]
Antiphospholipid syndrome	2.37 (0.56-10.11)
Atrial fibrillation/flutter	0.72 (0.35-1.49)
Cerebral venous sinus thrombosis/cerebrovascular accident/other	1.10 (0.28-4.35)
Venous thromboembolism	1.02 (0.51-2.04)

Abbreviation: PSM, patient self-management.

^a^ C-statistic = 0.692.

^b^ Reference is per 1-year increase.

### Implementation (Fidelity)

During the PSM phase, participants independently adjusted warfarin doses in response to out-of-range INRs in 83.0% of cases (366 of 441), mainly using their own experience (66.6% [243 of 365]) as opposed to using the online warfarin dosing tool (21.6% [79 of 365]) or other dosing decision support tools (11.8% [43 of 365]). Using personal experience for warfarin dosing was rated as very useful or useful by 97.4% of participants (113 of 116), compared with 74.1% (86 of 116) for the online tool and 56.9% (66 of 116) for other tools.

### Maintenance

At study conclusion, 98 participants (84.4%) preferred to continue PSM, 12 (10.3%) preferred clinic-based management, and 6 (5.2%) were unsure (eTable 3 in Supplement 1). Reasons for preferring continued PSM included confidence in dosing decisions from years of experience, access to anticoagulation clinicians if needed, greater autonomy, a sense of empowerment, and convenience. Reasons for preferring clinic-based management included greater comfort with clinician-led dosing, valuing regular clinician interactions, and less frequent INR monitoring. Clinicians were comfortable with continuing PSM for 109 participants (94.0%), uncomfortable for 3 (2.6%), and unsure for 4 (3.4%). Clinician discomfort with continuing PSM was attributed to frequent dosing needs, technical support requirements, missed INR checks, and poor communication.

## Discussion

In this single-group implementation trial, 120 participants at 4 US sites were successfully transitioned to PSM. Over half of those invited to participate in the PSM implementation study agreed to participate and provided consent. Of these, nearly 90% successfully transitioned to PSM. Even with excellent INR control at baseline, TTR improvements during the PSM phase were statistically significant. Over 80% of warfarin dose changes were made independently by participants without clinician input. Participants appeared confident in their ability to make warfarin dosing decisions, often using their own judgment rather than the provided clinical decision support tools. Clinical decision support tools may play a greater role for patients with less experience or less stable pre-PSM INR control. In prior work, we demonstrated that patients with TTR below 60% maintained improved TTR after transitioning to PSM when using dosing decision support tools.^33^ After transitioning to PSM, participants were more likely than clinicians to adjust weekly warfarin doses in response to out-of-range INRs. This may reflect clinicians’ higher tolerance for slightly out-of-range INRs. Bleeding and thromboembolic events were rare during PSM and did not differ from the 6 months prior to PSM or from patients who did not successfully transition. Of the 5 bleeding events recorded during PSM, the INR was elevated at the time of bleeding in 1 case. For the other 4 events, INR at the time of bleeding was not available; however, in 3 of these cases, the most recent INR prior to the event was within range or subtherapeutic. Bleeding events were reported to clinicians at the study site in 4 of 5 cases. These observations suggest US patients can independently manage warfarin dosing as effectively as their European counterparts. These findings also suggest an opportunity for anticoagulation clinics to optimize resource allocation. By transitioning appropriate patients to PSM, clinics may reduce routine management demands and redirect efforts toward higher-risk individuals and broader antithrombotic stewardship activities.

Although an estimated 4% of US patients own home INR monitors and the efficacy of PSM is well established, fewer than 1% are estimated to be engaged in PSM.^34^ This highlights significant potential for expanding PSM in US settings. Drawing on prior qualitative research that identified key barriers and facilitators to PSM, we developed the implementation toolkit described earlier. Recruitment focused on patients already using home INR monitors, as prior findings indicated this as a critical facilitator of successful PSM.^14,15^

Participants in this study were generally knowledgeable and experienced with warfarin therapy, reflected in high baseline AKT scores and the fact that most had been on warfarin for 2 or more years. Transition to PSM involved a spotters ready phase lasting 2 to 4 weeks, during which patients interpreted INR results and made dosing decisions under clinician supervision—either in person or via telehealth—until they felt confident managing PSM independently. We enrolled participants who were clinically stable and had high baseline TTR, which helped minimize the risk of INR volatility during PSM. During follow-up, no PSM participants were removed due to worsening health status or hospitalization. However, changes in disease status could impact safety and eligibility for PSM. Sites should have protocols in place to reassess PSM eligibility if patients experience significant clinical changes or hospitalization.

Our analysis found that age was the only factor independently associated with willingness to participate in the PSM implementation study, with older patients being less likely to participate. Clinicians at participating sites used their familiarity with potential candidates and input regarding patient competency and comfort with PSM to identify those most likely to engage with and succeed in PSM.

Unlike prior PSM trials, we observed minimal changes in warfarin knowledge, therapy satisfaction, and health-related quality of life during the PSM phase.^10^ This likely reflects differences in study design: earlier trials compared PSM with clinic-based management, whereas in our study, patients continued home INR testing but also assumed the added responsibility of independently adjusting their warfarin doses. Improvements in satisfaction and quality of life in prior trials may be more attributable to the shift to home monitoring than to self-management itself. Minimal changes in knowledge scores may be explained by participants’ extensive prior experience with warfarin—most had been on therapy for 2 or more years. Despite limited changes in these outcomes, patients overwhelmingly preferred the autonomy of PSM over relying on clinicians for dosing decisions after home INR testing. Clinicians, in turn, were comfortable with most patients continuing PSM at the study’s conclusion. Our PSM implementation study confirmed that with the right tools, training, and support, similar US patients can successfully engage in PSM.

Based on our study experience, we recommend the following strategies for implementing PSM. Initial recruitment should focus on patients with at least 2 years of warfarin experience who are already using home INR monitors. Candidate screening should be conducted through established patient-clinician relationships and the 5 screening questions described previously. PSM education should be delivered via an interactive online module. Transition to PSM should include the spotters ready phase until patients demonstrate sufficient confidence to assume full responsibility. Finally, patient and clinician comfort with PSM should be reassessed at regular intervals, typically every 1 to 3 months.

### Limitations

This study has several important limitations that should be considered when interpreting the results. Implementation sites were selected based on their Anticoagulation Forum Centers of Excellence credentials, which may limit generalizability. However, for this initial feasibility study, we intentionally partnered with organizations that demonstrated a supportive culture for PSM and a willingness to adapt workflows to accommodate its implementation. The enrollment target represented only about 2.5% of patients prescribed warfarin at participating sites, and the sample was predominantly White and non-Hispanic, further limiting generalizability. No patients receiving warfarin for management of a left ventricular assist device were included in the study. Additionally, restricting enrollment to clinically stable patients already using home INR monitors may have introduced selection bias. Nonetheless, prior research identified home monitoring as a key facilitator of successful PSM, and guidelines recommend PSM only for patients who can access and effectively use home testing equipment.^11,13^ We believed that focusing initial implementation efforts on this population would increase the likelihood of success. It is possible that patients using laboratory-based INR testing could also engage in PSM if they had timely access to results through a secure patient portal. Only participants who completed the PSM phase took the poststudy AKT, ACT, and SF-36 surveys, which may have introduced selection bias. The small number of consenting participants who did not transition to PSM limited our ability to model projections of successful transition, although we were able to identify older age as a factor associated with decreased willingness to participate. Other clinically relevant variables that may have influenced willingness to participate—such as having multiple morbidities, educational attainment, and other socioeconomic variables—were not collected. Although most patients preferred to continue PSM after the study, 2 of the 4 sites lacked policies to support PSM beyond the study period, resulting in a return to clinic-based management for those participants. Although the study was not powered to detect bleeding, thromboembolic, or mortality events, and the 6-month follow-up may have been insufficient for these outcomes, we observed a statistically significant increase in TTR—a surrogate marker associated with improved clinical outcomes.^32^ Throughout the study, clinicians remotely reviewed each warfarin dosing decision and intervened when necessary to prevent harm. While such oversight is not typical in some European PSM models, it likely helped build clinician confidence in patient decision-making. We were unable to report the exact prestudy duration of warfarin therapy, as the survey only captured whether patients had been on warfarin for less than 2 years or for 2 years or more; this limits interpretation of how prior experience may have influenced dosing decisions and study outcomes. Finally, the absence of a comparator group limits the robustness of our findings.

## Conclusions

The findings of this implementation trial demonstrated the feasibility of PSM in the US health care system and provide a roadmap for future PSM implementation efforts. Our study provides critically needed evidence to facilitate and accelerate PSM implementation and realization of increased safety for patients prescribed warfarin therapy in the US health care system.

## Notes

Supplement 1. eMethods. eTable 1. Participant responses to Anticoagulation Knowledge Tool (AKT), Anti-Clot Treatment Scale (ACTS), and Short Form-36 Questionnaires (SF-36) eTable 2. Baseline characteristics of consented participants by PSM transition status eTable 3. Agreement between participants and clinicians regarding continuing PSM at the end of the study

Supplement 2. Trial Protocol

Supplement 3. Data Sharing Statement
