# Supplementary material for: Warfarin Patient Self-Management in the US Health Care System: A Nonrandomized Clinical Trial
Source: JAMA Netw Open. 2026 Mar 19;9(3):e262627. doi: 10.1001/jamanetworkopen.2026.2627 (PMC13003370; doi:10.1001/jamanetworkopen.2026.2627)
Supplement: Supplement 1. — eMethods. eTable 1. Participant responses to Anticoagulation Knowledge Tool (AKT), Anti-Clot Treatment Scale (ACTS), and Short Form-36 Questionnaires (SF-36) eTable 2. Baseline characteristics of consented participants by PSM transition status eTable 3. Agreement between participants and clinicians regarding continuing PSM at the end of the study [file jamanetwopen-e262627-s001.pdf]

Supplemental Online Content

Witt DM, Hong H, Wilson AS, et al. Warfarin self-management in the US health care system. *JAMA Netw Open.* 2026;9(3):e262627. doi:10.1001/jamanetworkopen.2026.2627

eMethods.

eTable 1. Participant responses to Anticoagulation Knowledge Tool (AKT), Anti-Clot Treatment Scale (ACTS), and Short Form-36 Questionnaires (SF-36)

eTable 2. Baseline characteristics of consented participants by PSM transition status

eTable 3. Agreement between participants and clinicians regarding continuing PSM at the end of the study

This supplemental material has been provided by the authors to give readers additional information about their work.

© 2026 Witt DM et al. *JAMA Network Open*.

eMethods

Warfarin Patient Self-Management (PSM) Selection Criteria

- Identify patients to who would be a potentially good candidate for warfarin PSM.
- Eligibility for PSM is at the clinician's discretion.
- Some suggested parameters to consider when determining eligibility for this program include:
  - Is likely be compliant with:
    - Warfarin dosing
    - INR monitoring
    - Contacting and responding to clinicians as needed
  - Understands warfarin dose in number of milligrams, can calculate warfarin dose in milligrams per week and can identify the warfarin tablet strength used.
  - Willing to test and report the INR at least every 1 to 2 weeks.
  - Taking warfarin for at least 1 year.
  - Majority of health care provided within the system.
  - Able to obtain their INR result within 24 hours of testing (home INR monitor preferred).

PSM Educational Tools and Competency Assessment

https://warfarinpsm.s3.us-west-1.amazonaws.com/Warfarin+PSM+_Learning+Module+2026/story.html

Competency Assessment Questions (Bold=correct answer):

Let's say you are using 5 mg tablets, your current dose is 35 mg a week, INR goal is 2.0 to 3.0 and your INR today is 3.2. What should you do?

- Keep my warfarin dose the same and recheck INR in 1-2 weeks.
- **Decrease my dose by half a tablet to 32.5 mg a week and recheck INR in 1 week.**
- Decrease my dose by 2 half tablets to 30 mg a week and recheck INR in 1 week.
- Call my anticoagulation provider.

Let's say you are using 5 mg tablets, your current dose is 35 mg a week, INR goal is 2.0 to 3.0 and your INR today is 2.8. What should you do?

- **Keep my warfarin dose the same and recheck INR in 1-2 weeks.**
- Decrease my dose by half a tablet to 32.5 mg a week and recheck INR in 1 week.
- Increase my dose by half a tablet to 37.5 mg a week and recheck INR in 1 week.
- Call my anticoagulation provider.

Let's say you are using 5 mg tablets, your current dose is 35 mg a week, INR goal is 2.0 to 3.0 and your INR today is 2.8. You noticed some blood in your urine this morning, what should you do?

- Keep my warfarin dose the same and recheck INR in 1-2 weeks.
- Decrease my dose by half a tablet to 32.5 mg a week and recheck INR in 1 week.
- Increase my dose by half a tablet to 37.5 mg a week and recheck INR in 1 week.
- **Call my anticoagulation provider.**

© 2026 Witt DM et al. *JAMA Network Open*.

Let’s say you are using 3 mg tablets, your current dose is 27 mg a week, INR goal is 2.0 to 3.0 and your INR today is 1.5. What should you do?

- Keep my warfarin dose the same and recheck INR in 1-2 weeks.
- Increase my dose by half a tablet to 28.5 mg a week and recheck INR in 1 week.
- **Increase my dose by 2 half tablets to 30 mg a week and recheck INR in 1 week.**
- Call my anticoagulation provider.

Let’s say you are using 7.5 mg tablets, your current dose is 52.5 mg a week, INR goal is 2.0 to 3.0 and your INR today is 4.0. What should you do?

- Keep my warfarin dose the same and recheck INR in 1-2 weeks.
- Decrease my dose by half a tablet to 48.75 mg a week and recheck INR in 1 week.
- **Decrease my dose by 2 half tablets to 45 mg a week and recheck INR in 1 week.**
- Call my anticoagulation provider.

Let’s say you are using 7.5 mg tablets, your current dose is 52.5 mg a week, INR goal is 2.0 to 3.0 and your INR today is 1.6. The last 3 days your leg has been swollen, red, and tender when you touch it. What should you do?

- Keep my warfarin dose the same and recheck INR in 1-2 weeks.
- Increase my dose by half a tablet to 56.25 mg a week and recheck INR in 1 week.
- Increase my dose by 2 half tablets to 60 mg a week and recheck INR in 1 week.
- **Call my anticoagulation provider.**

Let’s say you are using 5 mg tablets, your current dose is 70 mg a week, INR goal is 2.0 to 3.0 and your INR today is 1.4. What should you do?

- Keep my warfarin dose the same and recheck INR in 1-2 weeks.
- Increase my dose by half a tablet to 72.5 mg a week and recheck INR in 1 week.
- Increase my dose by 2 half tablets to 75 mg a week and recheck INR in 1 week.
- **Call my anticoagulation provider.**

Let’s say you are using 5 mg tablets, your current dose is 70 mg a week, INR goal is 2.0 to 3.0 and your INR today is 2.5. You just finished an appointment with your orthopedic surgeon who would like to schedule you for a knee replacement in 6 weeks. What should you do?

- Keep my warfarin dose the same and recheck INR in 1-2 weeks.
- Increase my dose by half a tablet to 72.5 mg a week and recheck INR in 1 week.
- Increase my dose by 2 half tablets to 75 mg a week and recheck INR in 1 week.
- **Call my anticoagulation provider.**

Let’s say you are using 5 mg tablets, your current dose is 70 mg a week, INR goal is 2.0 to 3.0 and your INR today is 2.0. Your last 3 INRs were 2.8, 2.9, and 2.8 What could explain the drop in your INR (check all that apply)?

- **I missed a dose of warfarin.**
- **I started drinking a Premier Protein every day.**
- I drank a lot of extra alcohol.

© 2026 Witt DM et al. *JAMA Network Open*.

- I lost my appetite and didn't eat much.
- **I ate extra spinach this week.**

Approaches for Documenting PSM Activities in the Electronic Health Record

Sample Agreement for Patient Self-Management (PSM) of Warfarin Therapy:
This agreement outlines the expectations, responsibilities, and support provided to patients who choose to self-manage their warfarin therapy through the PSM program. The goal is to ensure safe, effective anticoagulation and ongoing partnership with your provider.

Patient Responsibilities

- INR Testing:
  - Test your INR every 1–2 weeks, or more frequently if advised during dose changes or instability.
  - Report all INR results promptly using the designated method (home meter or lab).
  - If your INR is less than 1.5 or greater than 5.0, contact your provider immediately.
- Warfarin Dosing:
  - Adjust your warfarin dose based on your INR results, following the education and protocols provided.
  - Keep a record of your dosing schedule and any changes made.
  - Contact your provider if you are unsure about dose adjustments or experience any issues.
- Communication:
  - Respond to messages or calls from your provider in a timely manner.
  - Call or message your provider when you need warfarin refills and include your current warfarin dose and tablet strength.
  - Notify your provider of any updates to your contact information.
  - Notify your provider of any hospitalizations, emergency room visits, upcoming surgeries or procedures, or significant bleeding/clotting concerns.
  - Participate in an annual review with your provider to discuss your self-management experience and review expectations.

Provider Responsibilities

- Education & Training:
  - Provide comprehensive training on warfarin dosing, INR testing, and self-management protocols.
  - Assess your readiness for PSM and support you through the transition period.
- Support & Oversight:
  - Review your INR results and self-management data regularly.
  - Contact you if there are concerns about your INR results, dosing, or compliance.
  - Discuss management during special situations such as hospitalizations, bleeding or clotting complications, surgeries, procedures, or interacting medications.
  - Decide to put PSM temporarily on hold due to complex health concerns or discontinue PSM altogether to ensure safe and effective warfarin therapy.
  - Offer ongoing education and answer questions as they arise.
  - Provide warfarin prescription refills.
- Annual Review:
  - Conduct an annual review of your PSM experience, reinforce education points and expectations, and address any concerns.
  - Provide feedback on your INR control, dosing, and overall safety.

© 2026 Witt DM et al. *JAMA Network Open*.

Sample INR Note for Patient Engaged in Warfarin Self-Management:
PSM (patient self-management of warfarin). Testing INR every 7-14 days. Is only communicating with provider if INR is outside 1.5-5.0, or if other concerns arise.
*Date of last phone call conversation or MyChart message reply: ***

Sample Provider Documentation of PSM Quality Measures Review:

- # INR tests: ***
- Time in Therapeutic Range (TTR)over the last 12-months: ***
- Mean INR (add all INR values and divide by number of tests): ***
- # INR >5: ***
- # INR <1.5: ***
- # INR's requiring clinic communication / intervention (Pharmacist or Technician)? ***
- # ED visits: ***
  - Any thrombosis or bleeding-related? {Yes/No}
- # Hospitalizations: ***
  - Any thrombosis or bleeding-related? {Yes/No}

Date of PSM initiation: ***
Date of last office visit with referring provider: ***
Does the patient have an active warfarin prescription? {Yes/No}
Number of INR tests outside of the desired interval of acceptable monitoring (# Late INR notices): ***

(OPTIONAL) Additional information: ***

<u>Assessment/Plan</u>
Patient's testing frequency, INR results and safety parameters indicate the patient's self-management of warfarin is {SATISFACTORY/UNSATISFACTORY}

Recommend {CONTINUE/DISCONTINUE} Warfarin PSM

Recommend next PSM review in *** months

Sample Patient Message Summarizing Review of PSM Quality Review:
As part of our ongoing quality assessment process for our warfarin self-management patients, we reviewed the last *** months of your INR results and clinical data as follows:

- You tested your INR *** times.
- The amount of time spent in your INR range of {INR goal options} over the last 12 months was ***%.
- Your average INR was ***.
- You had *** INRs greater than 5, and *** INRs less than 1.5.
- *** INRs were discussed with a provider from the clinic.
- You had *** bleeding or clotting events requiring ER or hospitalization.

Based on our review, it seems things are going well with your warfarin self-management. We will review again in *** months. Please give us a call if you have any questions or concerns or would like to discuss further.

Patient INR Survey Completed by Participants with Each INR Measured During PSM Phase

© 2026 Witt DM et al. *JAMA Network Open*.

Survey element	Response option(s)	Comment
1. What health system do you use for your warfarin?	-Brigham and Women's Hospital<br>-Loma Linda VAMC<br>-University of Michigan<br>-University of Utah	
2. What is your study ID?	-Patient entered assigned study ID number	
3. What is your goal INR range?	-2.0-3.0<br>-2.5-3.5	
4. Date of INR being reported?	-Patient entered date INR was performed	
5. Value of INR being reported?	-Patient entered INR value	If INR was 5.0 or higher, patient was instructed to contact their anticoagulation provider
6. Based on your INR value, do you plan on adjusting your weekly warfarin dose?	-Yes<br>-No	If "No" survey skipped to question 20
7. In response to this INR, who ultimately decided the new warfarin dose you will take?	-I did<br>-Someone else/healthcare provider<br>-Other	If "I did", question 8 was asked; otherwise, survey skipped to question 9
8. How did you decide which warfarin dose to take?	-My warfarin website tool<br>-My own decision<br>-Other	
9. Over the past week what tablet strength of warfarin did you use?	-1 mg (pink)<br>-2 mg (lavender)<br>-2.5 mg (green)<br>-3 mg (tan)<br>-4 mg (blue)<br>-5 mg (peach)<br>-6 mg (teal)<br>-7.5 mg (yellow)<br>-10 mg (white)<br>-I used different tablet strengths (more than one color)	
10. Over the PAST week, how many tablets did you actually take each day (enter 0 for missed doses)?	-Patient filled in a grid with the number of tablets taken each day	Dates for the grid were auto populated based on the INR date entered for question 4<br>Total milligrams taken over the previous week automatically calculated from values entered in the grid
11. Since your last INR, which of the following changes have occurred?	-Bleeding symptoms<br>-Clotting symptoms<br>-New or worsening illness<br>-Changes in medications	If "Bleeding symptoms" question 12 was asked<br>If "Clotting symptoms" question 13 was asked

© 2026 Witt DM et al. *JAMA Network Open*.

	-Changes in diet/vitamin K intake<br>-None	If “New or worsening illness” question 14 was asked<br>If “Changes in medications” question 15 was asked<br>If “Changes in diet/vitamin K intake” question 16 was asked<br>If “None” survey skipped to question 18
12. What bleeding symptoms (select all that apply)?	-Nosebleed<br>-Unusual bruising (i.e., more severe or more frequent than usual)<br>-Heavy menstrual bleeding<br>-Blood in urine (red or pink)<br>-Blood in stool (bright red or black)<br>-Coughing up blood<br>-Throwing up blood<br>-Stroke symptoms such as facial drooping, arm weakness, and/or speech difficulties<br>-Other	
13. What clotting symptoms (select all that apply)?	-New pain, redness, or swelling of legs or arms<br>-Chest pain (i.e., more severe or frequent than usual)<br>-Shortness of breath (i.e., more severe or frequent than usual)<br>-Stroke symptoms such as facial drooping, arm weakness, and/or speech difficulties<br>-Other	
14. What new or worsening illness symptoms (select all that apply)	-Fever or infection<br>-Vomiting (i.e., more severe or frequent than usual)<br>-Diarrhea (i.e., more severe or frequent than usual)<br>-Visit to urgent care<br>-Visit to emergency room<br>-Admitted to hospital<br>-Other	
15. What changes in medications (prescription, over the counter, and/or supplements) (select all that apply)?	-Started new medication(s)<br>-Stopped medication(s)<br>-Changed dose of medication(s)<br>-Other	Patients were asked to provide details of any changes

© 2026 Witt DM et al. *JAMA Network Open*.

16. What changes in diet/vitamin K intake (e.g., green leafy vegetables, vegetable smoothies, nutrition, or vitamin supplements with vitamin K)	-INCREASED vitamin K intake<br>-DECREASED vitamin K intake<br>-Other	
17. Did you contact your anticoagulation management service provider regarding these symptoms?	-Yes<br>-No	
18. Did you already take your warfarin dose for today before checking your INR?	-Yes<br>-No	Used to auto-populate dates of the grid for question 19
19. Over the NEXT week, how many tablets do you plan on taking each day?	-Patient filled in a grid with the planned number of tablets for each day	Total milligrams taken over the next week automatically calculated from values entered in the grid
20. When do you plan on rechecking your next INR?	-Patient entered date of next INR	
21. Is there anything else you wanted to tell us about today's INR?	-Text box for patient comments	

INR-international normalized ratio; PSM-patient self-management; VAMC-Veterans Administration Medical Center; ID-identification

© 2026 Witt DM et al. *JAMA Network Open*.

Examples of Warfarin Dosing Clinical Decision Support Tools:

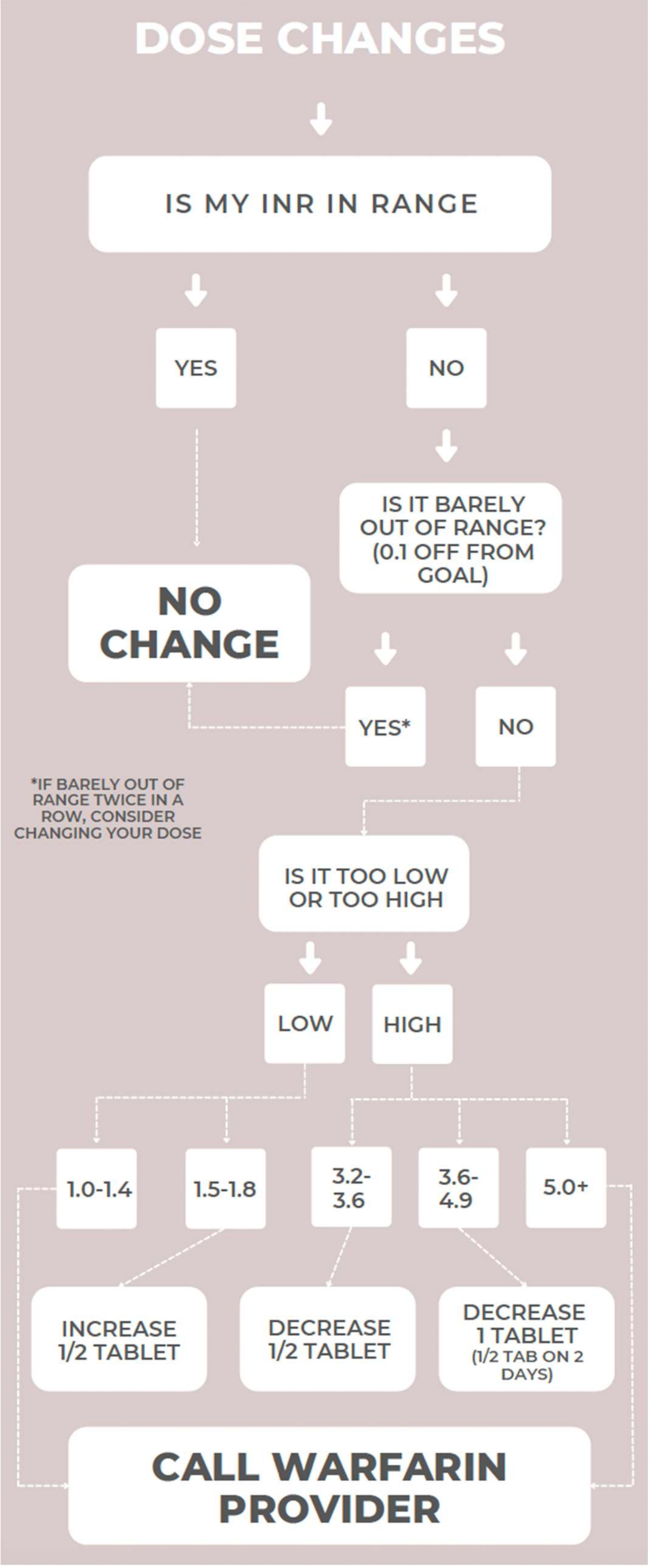

© 2026 Witt DM et al. *JAMA Network Open*.

Dosing changes using Tablets

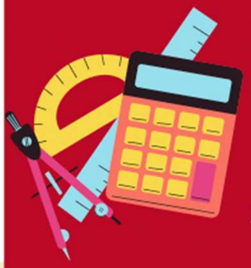

CALCULATE WEEKLY DOSE

1. Add number of tablets you take every day for one week
2. Multiply total # of tablets by the tablet strength

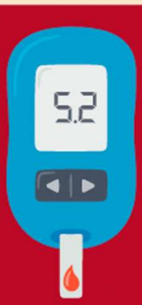

EVALUATE INR

If INR just out of range (1.9 or 3.1, you may not need to make a dose change.
If your INR is less than 1.5 or more than 5.0 please call your provider

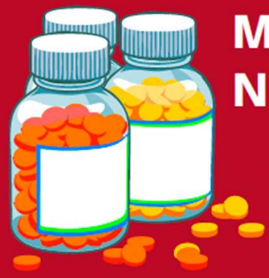

MAKE DOSE CHANGE IF NEEDED

1.5-1.8: Increase by 1/2 tablet per week
3.2-3.6: Decrease by 1/2 tablet
3.7-4.9: Decrease by 1 tablet (1/2 tablet on two different days)

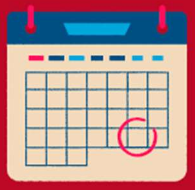

RECHECK INR IN 1-2 WEEKS

If your INR has been staying in range, you may choose to check in 2 weeks.
If your INR is out of range, always check in 1 week.

© 2026 Witt DM et al. *JAMA Network Open*.

eTable 1. Participant responses to Anticoagulation Knowledge Tool (AKT), Anti-Clot Treatment Scale (ACTS), and Short Form-36 Questionnaires (SF-36)

Scale (n=120)	Mean Baseline Score (SD) [range]	Change after PSM	p-value[a]	p-value[b]
AKT	88.7 (10.6) [31.4 – 100]	0.9	0.51	0.35
ACTS - Benefit Scale	12.7 (1.9) [6 – 15.0]	-0.2	0.16	0.21
ACTS - Benefit Global	4.0 (1.1) [1.0 – 5.0]	-0.1	0.32	0.35
ACTS - Burden Scale	52.2 (6.8) [27.0 – 60.0]	-0.5	0.07	0.09
ACTS - Burden Global	4.2 (1.0) [1.0 – 5.0]	0.1	0.53	0.65
SF-36 - Physical Functioning	65.5 (30.1) [0.0 – 100]	0.8	0.94	0.96
SF-36 - Role Limitations Due to Health	62.5 (42.8) [0.0 – 100]	1.1	0.58	0.44
SF-36 - Role Limitations Due to Emotion	77.2 (38.3) [0.0 – 100]	2.2	0.50	0.42
SF-36 - Energy/Fatigue	54.4 (22.4) [0.0 – 90.0]	-2.0	0.34	0.39
SF-36 - Emotional Wellbeing	78.8 (17.2) [12.0 – 100]	1.1	0.65	0.77
SF-36 - Social Functioning	78.5 (26.8) [0.0 – 100]	0.5	0.85	0.89
SF-36 - Pain	62.5 (27.1) [0.0 – 100]	0.1	1.00	0.95
SF-36 - General Health	58.3 (22.4) [5.0 – 100]	-1.2	0.20	0.19

[a] Wilcoxon Signed Rank test

[b] Data structured hierarchically to account for nesting by site

SD – Standard deviation; PSM – Patient self-management

© 2026 Witt DM et al. *JAMA Network Open*.

eTable 2. Baseline characteristics of consented participants by PSM transition status (N=138)

Characteristic	Failed PSM Transition (n=18)	Transitioned to PSM (n=120)	p-value
Mean age[a] (years, SD)	58.1 (15.3)	64.0 (12.7)	0.13
Female (n, %)	8 (44.4)	50 (41.7)	0.82
Race (n, %)			0.31
American Indian/Alaska Native	0 (0.0)	1 (0.8)	
Asian	0 (0.0)	2 (1.7)	
Black/African American	1 (5.6)	4 (3.3)	
Multiracial	1 (5.6)	0 (0.0)	
Native Hawaiian/Other Pacific Islander	0 (0.0)	1 (0.8)	
Unknown/Undeclared	0 (0.0)	5 (4.2)	
White	15 (83.3)	102 (85.0)	
Other[b]	1 (5.6)	5 (4.2)	
Hispanic/Latin Ethnicity (n, %)	2 (11.1)	13 (10.8)	0.97
Organization (n, %)			0.39
Brigham and Women's	2 (11.1)	28 (23.3)	
Loma Linda	5 (27.8)	25 (20.8)	
Michigan	6 (33.3)	24 (20.0)	
Utah	5 (27.8)	43 (35.8)	
Primary Indication for Anticoagulation (n, %)			0.02
Antiphospholipid Syndrome	2 (11.1)	7 (5.8)	
Atrial Fibrillation/Flutter	2 (11.1)	28 (23.3)	
Cerebral Venous Sinus Thrombosis	0 (0.0)	2 (1.7)	
Cerebrovascular Accident	1 (5.6)	0 (0.0)	
Mechanical Heart Valve	4 (22.2)	49 (40.8)	
Venous Thromboembolism	7 (38.9)	32 (26.7)	
Other[c]	2 (11.1)	2 (1.7)	
Years Receiving Warfarin Therapy (n, %)			0.60
< 2 Years	2 (11.1)	9 (7.5)	
≥ 2 Years	16 (88.9)	111 (92.5)	

[a] As of recruitment date
[b] Includes participants who selected 'Other'
[c] Includes hypercoagulable state, lupus anticoagulant, protein C deficiency, Fontan procedure

© 2026 Witt DM et al. *JAMA Network Open*.

eTable 3. Agreement between participants and clinicians regarding continuing PSM at the end of the study (N=116)

	**Provider comfortable with patient continuing PSM**			
**Patient prefers to continue PSM**	Yes	No	Unsure	Total
Yes	94 (81.0%)	3 (2.6%)	1 (0.9%)	98 (84.4%)
No	9 (7.8%)	0	3 (2.6%)	12 (10.3%)
Unsure	6 (5.2%)	0	0	6 (5.2%)
Total	109 (94.0%)	3 (2.6%)	4 (3.4%)	116[a]

[a] Responses missing for 4 patients

PSM – Patient self-management

© 2026 Witt DM et al. *JAMA Network Open*.
