# Supplementary material for: Warfarin Patient Self-Management in the US Health Care System: A Nonrandomized Clinical Trial
Source: JAMA Netw Open. 2026 Mar 19;9(3):e262627. doi: 10.1001/jamanetworkopen.2026.2627 (PMC13003370; doi:10.1001/jamanetworkopen.2026.2627)
Supplement: Supplement 3. — Data Sharing Statement [file jamanetwopen-e262627-s003.pdf]

Data Sharing Statement

Data
Additional Information: ClinicalTrials.gov ID NCT04766216
Data available: Yes
Data types: Deidentified participant data
How to access data: dan.witt@pharm.utah.edu
When available: With publication

Supporting Documents
Document types: None

Additional Information
Who can access the data: Researchers whose proposed use of the data has been approved.
Types of analyses: For research aimed at improving the safety of warfarin therapy.
Mechanisms of data availability: With a signed data access agreement.
